# Pirfenidone prevents acute kidney injury in the rat

**DOI:** 10.1186/s12882-019-1364-4

**Published:** 2019-05-08

**Authors:** Ixchel Lima-Posada, Francesco Fontana, Rosalba Pérez-Villalva, Nathan Berman-Parks, Norma A. Bobadilla

**Affiliations:** 1 Molecular Physiology Unit, Instituto de Investigaciones Biomédicas, Universidad Nacional Autónoma de México, Vasco de Quiroga No. 15, Tlalpan, 14000 Mexico City, Mexico; 2 Department of Nephrology, Instituto Nacional de Ciencias Médicas y Nutrición Salvador Zubirán, Mexico City, Mexico; 3 Surgical, Medical and Dental Department of Morphological Sciences, Section of Nephrology, University of Modena and Reggio Emilia, Modena, Italy

**Keywords:** Ischemia/reperfusion injury, Hsp72, Nitric oxide

## Abstract

**Background:**

Pirfenidone is an orally active drug used for the treatment of idiopathic pulmonary fibrosis to slow loss of lung function; it acts mainly through an antifibrotic effect but also possesses antioxidant and anti-inflammatory properties. We assessed the effect of prophylactic administration of pirfenidone on acute kidney injury due to bilateral renal ischemia.

**Methods:**

Eighteen rats were included and divided in: 1) sham-operated rats (S), 2) rats underwent bilateral renal ischemia for 20 min (I/R), and 3) rats treated with pirfenidone 700 mg/kg/day 24 h before surgery and subjected to bilateral renal ischemia for 20 min (I/R + PFN). All the rats were euthanized and studied 24 h after renal reperfusion.

**Results:**

As was expected, the I/R group exhibited a significant reduction in creatinine clearance, urinary output and renal blood flow, as well as extensive tubular injury. These alterations were associated with a significant decrease in urinary excretion of nitrites and nitrates (UNO_2_/NO_3_V). In the I/R + PFN group, recovery of renal function and UNO_2_/NO_3_V was observed, together with lesser histological signs of tubular injury compared to the I/R group.

**Conclusions:**

This study shows that prophylactic administration of pirfenidone prevented acute kidney injury due to bilateral ischemia in the rat. Recovery of NO production appears to be one of the mechanism of pirfenidone renoprotective effect. Our findings suggest that pirfenidone is a promising drug to reduce renal injury induced by I/R.

## Background

Acute kidney injury (AKI) affects 21% of hospitalized patients, and the incidence is higher in the intensive care unit (60%) [1]. The most frequent causes of AKI are associated with generalized or localized ischemic damage due to surgery, sepsis, trauma, infections, dehydration and toxic drug damage [2]. AKI is often caused by a reduction in renal blood flow (RBF) that induces endothelial and tubular epithelial damage [3–7]. The reduced RBF causes a decrease in the perfusion of the peri-tubular capillaries, injuring the S2 and S3 segments of the proximal tubule, due to the large number of mitochondria that these segments possess. Therefore, the proximal tubule is highly susceptible to changes in oxygen tension, and the Na^+^/K^+^ ATPase pump is one of the most affected molecules [7–9]. In addition, the reduction of ATP produces an uncoupling in the respiratory chain with the subsequent formation of free radicals that encourages the detachment of epithelial cells and death by apoptosis or necrosis It is then reasonable that the resulting tubular damage is proportional to the severity of the ischemic event [10]. Paradoxically, these alterations are enhanced during renal reperfusion, which conditions a greater pro-oxidant and pro-inflammatory environment [11–13].

Endothelial cells also undergo several alterations, such as loss of their intercellular junctions, alterations in the cytoskeleton and endothelial glycocalyx, increased permeability of the microcirculation and interstitial edema. This, in turn, leads to an alteration in the supply of oxygen, which intensifies renal damage after decreased RBF. Simultaneously, there is an increase in the expression of adhesion molecules that allow inflammatory cells to adhere to the endothelium, with consequent leukocyte infiltration in the renal interstitium [14].

Although the tubular epithelium possesses the ability to regenerate, the cellular processes in endothelial and tubular cells may not recover completely; therefore, AKI is a risk factor for developing chronic kidney disease (CKD) [7, 15–18]. In a recent meta-analysis that included patients who survived an AKI episode, the reported incidences of chronic kidney disease (CKD) and end-stage renal disease (ESRD) were 25.8 and 8.6%, respectively [19]. These observations have greater relevance in the study carried out in 126 children who suffered AKI without any other pathophysiological condition, revealing that 10% of these children developed CKD in a period between 1 and 3 years [20]. Therefore, it is imperative to have efficient pharmacological treatments to prevent AKI.

Pirfenidone is an orally available pyridone analog (5-methyl-1-phenyl-1*H*-pyridin-2-one) approved for clinical use in idiopathic pulmonary fibrosis [21]. Indeed, it has been reported in several animal models of progressive fibrotic disorders that this compound not only displays antifibrotic activity, but also anti-inflammatory and antioxidant activities [22–32]. Specifically, in experimental models of renal injury such as subtotal nephrectomy, hypertension, calcineurin inhibitor nephropathy, and diabetic nephropathy, pirfenidone administration reduced proteinuria, lowered the rate of loss of glomerular filtration rate (GFR), decreased interstitial fibrosis and macrophage infiltration, improved mitochondrial dysfunction and reduced mesangial matrix expansion [26–28, 31–33]. Supporting this, a randomized, double-blind, placebo-controlled study that was performed in subjects with diabetic nephropathy found that after one year of therapy, the estimated glomerular filtration rate (eGFR) increased significantly in the pirfenidone-treated group, whereas in the placebo group it was reduced even more [30]. In another small trial in patients with focal and segmental glomerulosclerosis, pirfenidone was able to reduce the rate of loss of eGFR [25].

Although pirfenidone is known to possess a consistent antifibrotic effect, its antioxidant and anti-inflammatory properties allowed us to postulate that pirfenidone could be effective in reducing renal injury induced by ischemia/reperfusion.

## Methods

All experiments containing animals were conducted in accordance with the recommendations in the Guide for the Care and Use of Laboratory Animals of the National Institute of Health. The protocol was approved by the Committee on Ethics of Instituto Nacional de Ciencias Médicas y Nutrición Salvador Zubirán. All surgeries were performed under sodium pentobarbital anesthesia, and all efforts were made to minimize suffering.

### Experimental protocol

Eighteen male Wistar rats weighing 270–300 g (age: 10 to 12 weeks old) breeding and housing in our animal facility (the colony was purchased in Charles River Laboratories, Raleigh, NC, USA) were included. The rats were divided in three groups: 1) sham-operated rats (Sham, *n* = 6), 2) rats subjected to bilateral renal ischemia of 20 min without any pharmacological prophylaxis (I/R, *n* = 6), and 3) rats administered pirfenidone 700 mg/kg/day 24 h before surgery and subjected to bilateral renal ischemia of 20 min (I/R + PFN, *n* = 6). We decided to use the minimum rats necessary in order to comply with the principle of the 3R (Replacement, Reduction and Refinement), in special with the “Reduction” that implies the design of methods that minimize the number of animals used per experiment. Moreover, the physiological variability of rats is lesser than mice, allowing to use a smaller number of animals per group.

Pirfenidone was provided by Cell Pharma. Standard rat diet was powdered and the pirfenidone was homogeneously incorporated in it to reach the desired dose. The amount of food consumed daily by rats, according to age and weight, was previously determined by observation of a dedicated group of rats (approximately 20 g/day). The required dose of Pirfenidone was mixed with the amount of food that rats would consume in 24 h, in order to ensure appropriate drug exposure. The used dose of pirfenidone (700 mg/kg/day) was previously reported by Ji X, et al. and Takakuta K, et al. [26, 33]. We acknowledge that half-life of Pirfenidone is quite short, and the desired dose was consumed by rats in the 24 h that preceded the I/R in a non-clearly determinable fashion. Nevertheless, since 80% of the dose of Pirfenidone is excreted by the urine, systemic exposure is substantially increased with severely impaired kidney function. Control and IR groups received the reconstructed pellets but without pirfenidone.

All rats were allowed to acclimate in metabolic cages three days before the experiment. The rats were euthanized and studied 24 h after sham surgery or reperfusion. A sham operated group treated with PFN was not included because the 3R’s principle and because pirfenidone did not modify the basal renal function as has been previously reported [34].

### Ischemia/reperfusion model

Experimental and control groups were anesthetized with an intraperitoneal injection of pentobarbital sodium (30 mg/kg) and sited on a heating pad to maintain a constant temperature at 37 °C, that was monitored with a rectal thermometer. A midline abdominal incision was made, and both kidneys were exposed. Renal ischemia was induced by non-traumatic vascular clamp over the isolated renal pedicles, [35–46], for 20 min in the rats corresponding to the I/R and I/R + PFN groups. Thereafter, the clamps were released to alloow reperfusion. Sham-operated animals underwent anesthesia, laparotomy, and renal pedicle dissection only. The incision in the muscle and the skin were closed with 3–0 vicryl and silk sutures, respectively.

### Biochemical studies

Two hours after renal ischemia, rats were placed in metabolic cages at 22 °C with a 12:12-h light-dark cycle and allowed free access to water. Individual 24-h urine samples were collected. Urine and serum creatinine concentrations were determined with Quantichrom creatinine assay kit (DICT-500), and concentration was determined in an ELISA reader (BioTek®, Power Wave XS2), and renal creatinine clearance (CrCl) was calculated by the standard formula C = (UxV)/P, where U is the concentration in urine, V is the urine flow rate, and P is the serum concentration. Microalbuminuria was measured by Beckman Coulter analyzer UniCel® DxC600 Synchron® Clinical System and the microalbuminuria-creatinine ratio was calculated.

### Functional parameters

Twenty-four hours after renal I/R, rats were anesthetized with an intraperitoneal injection of sodium pentobarbital (30 mg/kg) and placed on a heating pad to maintain a constant temperature at 37 °C, monitored with a rectal thermometer. The mean arterial blood pressure (MAP) was monitored with a transducer of pressure (model p23 db, Gould) by catheterized femoral arteries with polyethylene tubing (PE-50) and recorded with a polygraph (Grass Instruments, Quincy, MA). To recorded the renal blood flow (RBF), a midline abdominal incision was made to expose and dissect the left renal artery, and then an ultrasound transit-time flow probe (1RB, Transonic, Ithaca, NY) was placed around it and filled with ultrasonic coupling gel (HR Lubricating Jelly, Carter- Wallace, New York, NY). At the end of the experiment, the right kidney was removed, and the cortex and medulla were isolated, frozen in liquid nitrogen and stored at − 70 °C for molecular studies.

### Histopathological studies

The left kidney was perfused through the femoral catheter with saline solution, thereby preserving the mean arterial pressure of each animal. Following blanching of the kidney, the perfusate was replaced by a freshly prepared solution of formaldehyde 4% until fixation was completed, monitoring the mean blood pressure that each rat had during the experiment. At the end of the experiment, rats were killed with and overdose of intraperitoneal injection of sodium pentobarbital (120 mg/kg).

After appropriate dehydration, kidney slices were embedded in paraffin, sectioned at 3 μm and stained via the periodic acid-Schiff technique (PAS). Ten subcortical and juxtamedullary fields were recorded from each kidney slide with a digital camera integrated with a Nikon microscope; digital microphotographs were recorded for each rat at magnification × 100. Researchers who were blind to the experimental conditions analyzed preparations. Tubular injury was assessed by counting the number of casts per field and the number of damaged tubules per field. Tubular damage was characterized by a loss of brush border, detachment of cells from basement membrane, and lumen dilatation or collapse.

### Urinary Hsp72 levels

The Hsp72 levels in urine were assessed by Western blot analysis as a marker of renal injury as we have previously reported [37–40, 42–47]. The proteins in 1 μl of each urine were electroforetically separated in an acrylamide gel and transferred into a PVDF membrane (dilution 1:10). The membranes were incubated with anti-Hsp72 antibody (ENZO Life Sciences, 1:5000) overnight. Thereafter, membranes were incubated with a secondary antibody, HRP-conjugated goat anti-mouse IgG (1:5000, Santa Cruz Biotechnology) at room temperature. The proteins were detected using a chemiluminescence kit (Millipore). The densitometric analysis was performed in a UVP EC3 Imaging System for Image acquisition and analyzed with UVP Vision Works LS Analysis Software.

### Urinary nitrites and nitrates excretion (UNO_2_/NO_3_V)

NO production was indirectly determined in the urine samples from each rat using a colorimetric Nitric Oxide Assay Kit (Oxford Biomedical Research, Inc., Oxford, MI). In this assay, the sample nitrates are reduced to nitrites through nitrate reductase, followed by nitrite quantification using Griess reagent and detection at 540 nm. The urinary NO level was expressed in total μmoles per 24 h.

### Renal endothelial nitric oxide synthase (eNOS), catalase (CAT), and glutathione peroxidase (GPX) mRNA levels

The total RNA was extracted from frozen renal cortex by using the TRIzol method (Invitrogen, Carlsbad, CA). The integrity of the RNA was checked in an 1% agarose gel electrophoresis. Total RNA samples were treated with DNAase (DNAase I; Invitrogen) to avoid DNA contamination. Reverse transcription (RT) was carried out by using one μg of total RNA incubated for 1 h at 37° with M-MLV (Invitrogen). The mRNA levels of eNOS, catalase, and GPX were determined by real-time PCR on an ABI Prism 7300 Sequence Detection System (TaqMan, ABI, Foster City, CA). mRNA levels were determined by using the following probes: eNOS (Rn02132634_s1), CAT (Rn00560930_m1), and GPX (Rn00577994_g1). As a control, eukaryotic 18S rRNA was used (Rn03928990_g1). The comparative threshold cycle (Ct) method was used to determine the relative quantification of the expression of each gene.

### Statistical analysis

The results are presented as the means ± SD. The significance of the differences between groups was assessed by ANOVA using the Bonferroni correction for multiple comparisons. Statistical significance was defined as when the *p* value was < 0.05.

## Results

**Fig. 1:**
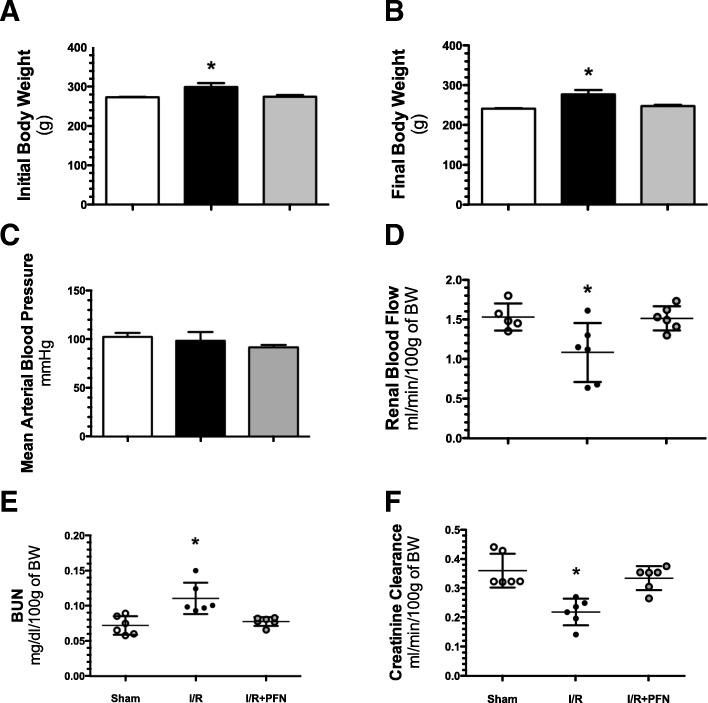
Renal dysfunction is prevented by pirfenidone. **a**) Initial body weight, **b**) final body weight (**c**) mean arterial blood pressure, (**d**) renal blood flow, (**e**) BUN and (**f**) creatinine clearance. The sham group is represented by white bars or dots, the I/R group by black bars or dots and the IR + PFN group by gray bars or dots. Six rats per group were studied. Data are shown as the means ± SD. * = *p* < 0.05 vs. sham and I/R + PFN groups. The significance of the differences between groups was assessed by ANOVA using the Bonferroni correction for multiple comparisons

The physiological parameters evaluated 24 h after surgery are presented in Fig. 1. The mean body weight (BW) was slightly higher in the I/R group, but this increase was according to initial body weight that was slightly higher (Fig. 1a and b). The mean arterial blood pressure was similar among the studied groups (Fig. 1c). The renal injury induced by I/R was evidenced by the significant reduction in renal blood flow and creatinine clearance, together with a significant elevation of BUN, compared to the sham group. Thus, renal blood flow in the I/R and sham groups was 1.1 ± 0.4 and 1.5 ± 0.2 ml/min/100 g of BW, respectively, (*p* < 0.05) (Fig. 1d); for BUN, the mean values were 30.4 ± 3.3 and 0.1 ± 0.01 (*p* < 0.05), respectively (Fig. 1e); and for creatinine clearance, the mean values were 0.2 ± 0.04 and 0.4 ± 0.06 (*p* < 0.05), respectively (Fig. 1f). In contrast, these three renal hemodynamic alterations were not seen in the I/R + PFN group, which showed a restoration of kidney function. The mean values for renal blood flow, BUN and creatinine clearance were 1.5 ± 0.2, 0.07 ± 0.002 and 0.3 ± 0.04 (p = NS), respectively (Fig. 1d, e and f). But, these mean values were significantly different respect to the I/R group (*p* = 0.0003, *p* = 0.001 and *p* = 0.004, respectively).

**Fig. 2:**
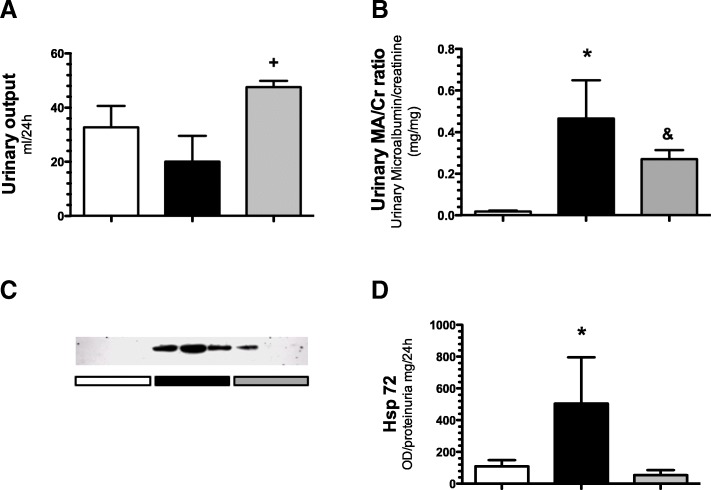
Renal structural injury induced by ischemia/reperfusion was prevented by pirfenidone administration. **a**) Urinary output, **b**) urinary microalbumin/creatinine ratio (MA/Cr), **c**) urinary Hsp72 levels by Western blotting and **d**) densitometric analysis of urinary Hsp72 levels normalized by proteinuria. The sham group is represented by white bars; the I/R group, by black bars; and the IR + PFN group, by gray bars. Six rats per group were studied. Data are shown as the means ± SD. * **=** *p* < 0.05 vs. sham and I/R + PFN groups, + **=** *p* < 0.05 vs. I/R, & **=** *p* < 0.05 vs. sham group. The significance of the differences between groups was assessed by ANOVA using the Bonferroni correction for multiple comparisons

In accordance with the acute kidney injury (AKI) induced by I/R, there was a significant reduction in urinary output compared to the sham group (20 ± 9.5 vs. 32.7 ± 19.3 ml/day, respectively (*p* < 0.05); in contrast, the I/R + PFN group did not exhibit a reduction in urinary output (47.5 ± 5.7 ml/day), as shown in Fig. 2a. The urinary micro-albumin/creatinine ratio increased significantly in the I/R group compared to control group. An effect that was partially prevented by pirfenidone (Fig. 2b). To indirectly evaluate the tubular injury, the urinary levels of heat shock protein 72 (Hsp72) were evaluated in the studied groups. The higher extent of AKI in the I/R group and the renoprotection conferred by pirfenidone was evidenced by the Western blot analysis for urinary Hsp72 levels, which were strongly positive in the I/R group, while they were very low or undetectable in the I/R + PFN group (Fig. 2c and d).

**Fig. 3:**
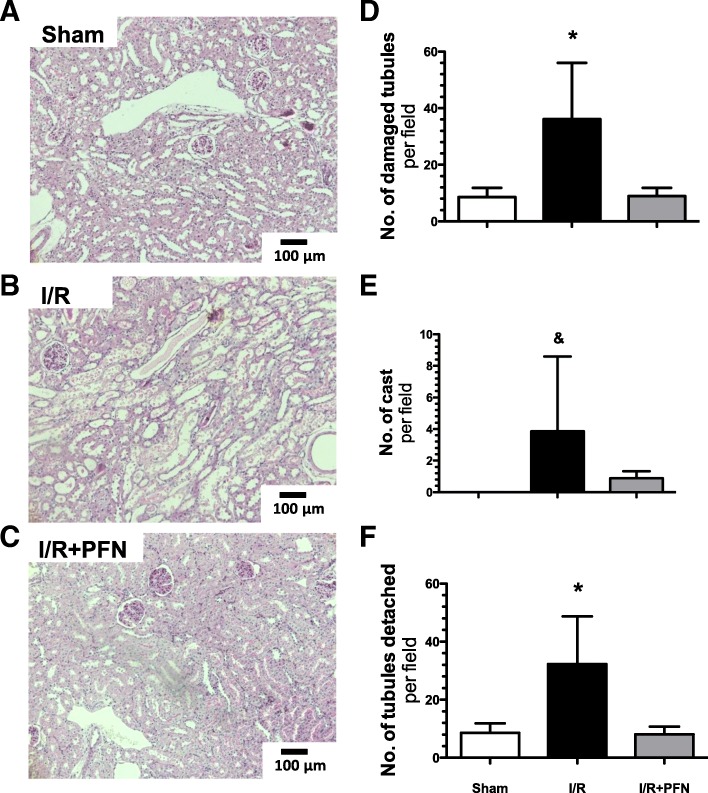
Histological changes induced by renal ischemia/reperfusion are prevented by pirfenidone. **a**-**c**) Representative light microphotographs of kidney slides stained with PAS from sham, I/R, and IR + PFN groups (× 100 magnification). **d**) Quantification of damaged tubules, **e**) number of casts formed, **f**) number of detached tubules. The sham group is represented by white bars; the I/R group, by black bars; and the IR + PFN group, by gray bars. Six rats per group were studied. Data are shown as the means ± SD. * = *p* < 0.05 vs. sham and I/R + PFN groups and & = *p* < 0.05 vs. sham group. The significance of the differences between groups was assessed by ANOVA using the Bonferroni correction for multiple comparisons

Representative light microscopy sections from the kidneys of sham, I/R and I/R + PFN groups stained with periodic acid–Schiff (PAS) are shown in Fig. 3a-c, respectively. The histopathological analysis revealed that the injury induced by I/R was characterized by tubular dilation, brush border loss, cell tubular detachment and cast formation (Fig. 3b). All of these alterations were absent in pirfenidone-treated rats (Fig. 3c). Accordingly, the damaged tubules per field were significantly higher in the I/R group than in the other two groups (Fig. 3d); the same happened with the number of casts per field, but statistical significance was only reached in comparison to the sham group (Fig. 3e). Similarly, the number of tubular cells detached from tubules was significantly higher in the I/R group when compared to the other two groups (Fig. 3f).

**Fig. 4:**
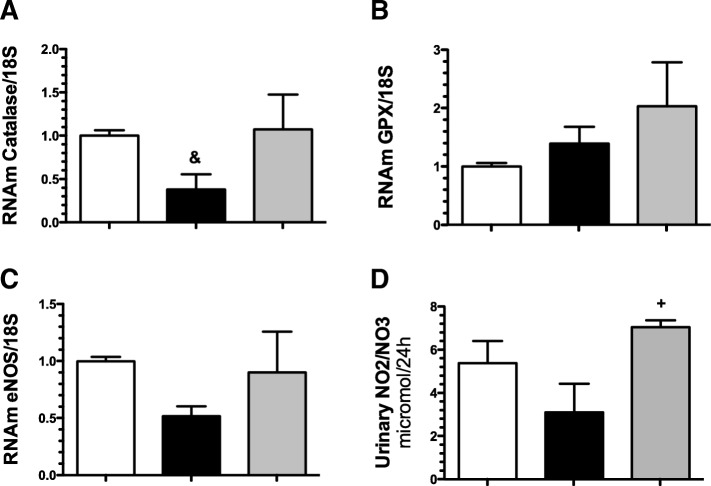
Mechanisms involved in renoprotection conferred by pirfenidone. **a**) Catalase mRNA levels, **b**) GPX mRNA levels, **c**) eNOS mRNA levels, **d**) urinary NO2/NO3 excretion The sham group is represented by white bars; the I/R group, by gray bars; and the IR + PFN group, by black bars. Six rats per group were studied. The results are presents as mean of two different measurements. Data are shown as the means ± SD. + = *p* < 0.05 vs. I/R, & = *p* < 0.05 vs. sham group. The significance of the differences between groups was assessed by ANOVA using the Bonferroni correction for multiple comparisons

We also evaluated the mRNA levels of several signaling pathways involved in the pathophysiology of AKI. Figure 4a and b show the mRNA levels of two antioxidant enzymes: catalase and glutathione peroxidase (GPx). Catalase was significantly reduced in the I/R group (Fig. 4a), but GPx mRNA levels remained unaltered among the groups (Fig. 4b); additionally, we measured the SOD levels, but no differences were found (data not shown). As reported previously, the renal hypoperfusion induced by I/R was associated with a reduction of endothelial nitric oxide synthase (eNOS) mRNA levels, but not reach the level of statistical significance by ANOVA, an effect that was not seen in the I/R + PFD group (Fig. 4c). Accordingly, urinary NO_2_/NO_3_ excretion was decreased in the I/R group, 3.1 ± 1.3 vs 5.4 ± 2.5 μmol/24 h in the sham group, but the difference was not significant by ANOVA. Interestingly, the urinary NO_2_/NO_3_ excretion was restored in the I/R + PFN group (7.05 ± 0.78 μmol/24 h, *p* < 0.05) as depicted in Fig. 4d.

## Discussion

Several studies conducted in experimental models and in humans have clearly demonstrated that pirfenidone possesses antifibrotic, antioxidant and anti-inflammatory properties [22–32]. Little is known, however, if its antioxidant and anti-inflammatory could be beneficial after an episode of acute kidney injury.

Our results shows that that pirfenidone confers protection against AKI induced by bilateral renal ischemia in rats. As expected, the I/R group exhibited a significant decrease in renal blood flow, creatinine clearance, and urinary output. All these alterations were accompanied by a significant reduction in urinary NO metabolites. In our previous reports, the degree of renal injury induced by 20 min of bilateral renal ischemia was similar to this study, in spite of the renewal of the breeding stock in our animal facilities [35, 37, 38, 48]. In contrast, pirfenidone-treated rats showed normal kidney function even when they had a similar degree of ischemic injury. Confirming these findings, extensive histological signs of tubular injury, also evidenced by the reduction in Hsp72 urinary levels.

Acute kidney injury induced by I/R is a multistep process which starts with the hypoperfusion phase, leading to hypoxia and inflammation, as well as increased reactive oxygen species. Renal blood flow reduction, which is responsible for the initiation of the process [49], that later also contributes to the extension of kidney injury. Vasomotor tone in the kidney is mainly controlled by NO derived from eNOS. It has been demonstrated that the effect of NO derived from eNOS in glomerular afferent arterioles is protective against I/R injury [50], while NO produced by inducible nitric oxide synthase (iNOS) may contribute to tubular ischemic injury. Decreased eNOS function characterizes endothelial dysfunction associated with AKI [51], while increased eNOS activity induced by ischemic preconditioning or inhibition of Rho kinase in rats could protect the kidneys from I/R injury [52, 53]. Thus, the production of NO by eNOS appears to have a protective effect in the setting of ischemic AKI. In this study, we found that I/R injury was associated with a reduction in urinary NO_2_/NO_3_ excretion after 24 h of ischemia. Interestingly, the restoration of renal blood flow in the I/R + PFN group was associated with the restoration of urinary NO_2_/NO_3_ excretion to values comparable to those observed in the sham group. This finding supports the hypothesis of an immediate antioxidant effect of pirfenidone, possibly mediated by the improvement in NO generation after the ischemic insult; this effect of pirfenidone is likely to be responsible for the preservation of renal function in the I/R + PFN group.

Renal NO generation was confirmed by the analysis of eNOS mRNA levels. Indeed, eNOS mRNA levels were lower in the I/R group when compared to the control groups, but this downregulation was not detected in the I/R + PFN group, suggesting a better response in eNOS activity after an AKI episode by pirfenidone administration.

It is well known that GPX and CAT are enzymes with peroxidase activity that are responsible for protection from oxidative damage; both convert the reactive oxygen species in water and oxygen, thereby mitigating its toxic effects. Activation of CAT after the blockade of angiotensin II receptor has been associated with beneficial effects in the hypertensive and post-ischemic kidney [54], and higher serum levels of GPX have been reported in a pharmacological model capable of attenuating renal I/R injury [55]. In accordance with this evidence, we observed a significant reduction in catalase mRNA levels in the I/R group compared with the control group, while in the I/R + PFN group, catalase mRNA levels were similar to those of the control group. When we analyzed GPX mRNA levels, differences among the groups did not differ significantly, but a tendency towards increased expression in the I/R + PFN group was observed. The improved antioxidant response in the pirfenidone-treated rats compared with I/R group might be due to the antioxidant activity of pirfenidone and contribute to the explanation of its protective effect in ischemic AKI.

Many studies reported an antiproteinuric effect for pirfenidone [26, 27, 33]. Although they were all based on chronic models of kidney disease, where proteinuria is both an indicator of tissue damage and a risk factor for disease progression. Moreover, some authors [56] recognized that the effect of pirfenidone in proteinuria reduction was small. In this acute study of renal injury, we found that pirfenidone was able to reduce the urinary microalbumin/creatinine ratio compared to the I/R group indicating the potential effect of pirfenidone in reducing ischemic injury. This was also confirmed by lesser tubular damage and urinary Hsp-72 excretion, together with, the improvement in creatinine clearance and BUN.

In general, pharmaceutical products cannot be prescribed prophylactically for the treatment of AKI, and the disease itself is not very predictable; for this reason, the beneficial effects of pirfenidone could be difficult to detect in a clinical setting. However, the preliminary results of this study encourage future experiments that may shed more light on the renoprotective mechanisms of pirfenidone in the context of AKI.

## Conclusion

In summary, we show that prophylactic administration of pirfenidone protects the kidney against I/R injury by preventing renal dysfunction and structural damage. The mechanism of renoprotection appears to involve the preservation of renal blood flow possibly secondary to the restoration of NO production. These results may contribute to the research into new therapeutic solutions for the prevention of AKI in patients who are expected to be exposed to renal I/R injury, such as kidney transplant recipients and patients undergoing high-risk cardiovascular surgery.

## Abbreviations

AKI: Acute kidney injury
BW: Body weight
CAT: Catalase
CKD: Chronic kidney disease
CrCl: Creatinine clearance
eGFR: Estimated glomerular filtration rate
eNOS: Endothelial Nitric Oxide Synthase
ESRD: End-stage renal disease
GFR: Glomerular filtration rate
GPX: Glutathione Peroxidase
Hsp72: Heat shock protein 72
I/R: Ischemia reperfusión
iNOS: Inducible nitric oxide synthase
MAP: Mean arterial blood pressure
NO: Nitric oxide
PAS: Periodic acid-Schiff
PFN: Pirfenidone
RBF: Renal blood flow
UNO_2_/NO_3_V: Urinary excretion of nitrites and nitrates
